# Morphology, Phylogeny and Pathogenicity of *Colletotrichum* *menglaense* sp. nov., Isolated from Air in China

**DOI:** 10.3390/pathogens10101243

**Published:** 2021-09-26

**Authors:** Min Qiao, Jie Li, Lin-lin Fang, Jian-ying Li, Ze-fen Yu

**Affiliations:** 1 Laboratory for Conservation and Utilization of Bio-resources, Key Laboratory for Microbial Resources of the Ministry of Education, Yunnan University, Kunming 650091, China; qiaoming@ynu.edu.cn (M.Q.); lijie951021@163.com (J.L.); fllcsz@mail.ynu.edu.cn (L.-l.F.); 2 Kunming Edible Fungi Institute of All China Federation of Supply and Marketing Cooperatives, Kunming 650221, China; jianying0818@163.com

**Keywords:** airborne fungi, coelomycetes, *Colletotrichum gloeosporioides* complex, taxonomy, pathogenicity

## Abstract

A new species, *Colletotrichum menglaense*, isolated from air in Mengla, Xishuangbanna, Yunnan Province, China, was characterized and described combining morphological characteristics and multigene phylogenetic analysis. Morphologically, it is characterized by oblong, sometimes slightly constricted, micro-guttulate conidia and simple obovoid to ellipsoidal appressoria. Phylogenetic analysis of the ITS, ACT, CHS, and GAPDH sequences showed that *C. menglaense* belongs to the *C. gloeosporioides* complex. The pathogenicity of *C. menglaense* on fruits of several crop plants, including strawberry, orange, grape, tomato, and blueberry, was tested and confirmed by the re-isolation of *C. menglaense*.

## 1. Introduction

There are numerous bacteria and fungi in the near-surface atmosphere and upper troposphere, with millions of cells per cubic metre of air [1]. More than 40,000 species of fungi live in the air, mainly from soil, animals and plants, human activities, and excreta [2]. Fungi eject spores into the atmosphere by water jets or droplets, and become an important part of aerosols [3,4]. Hence, airborne fungi are closely related to air pollution, environmental quality, and human health.

Earlier investigations of microorganisms in air were connected with dust [5,6] or aerosol particles [7,8,9,10,11] to detect the fungal distribution or characterisation. Other studies suggested that a considerable proportion of indoor airborne fungi were derived from outdoor fungi [12,13], and fungi had a great impact on human health [14,15,16,17,18]. With the advance of molecular approaches and the awareness of the importance of mycobiota diversity research, the number of studies about outdoor mycobiota is increasing. Many investigations recovered fungi from various places: the atmosphere in the urban environment of the USA [19], a rural area in India [20], at an altitude of 20,000 m in Earth’s atmosphere [5], in southeastern Austria [16], and from the Himalayan region [21]. Most of these studies monitored remote, extreme, sparsely populated sites to learn the diversity, distribution, and seasonal variations of fungal species [22]. Several genera were often detected in air samples, *Curviclavula* G. Delgado et al. [23], *Aspergillus* P. Micheli, *Penicillium* Link, and *Talaromyces* C.R. Benj [13], and occasionally also species of *Colletotrichum* Corda [24,25].

*Colletotrichum* (Sexual morph: *Glomerella*) is a genus of the family Glomerellaceae, Glomerellales, Sordariomycetes, which was established with *C. lineola* Corda as the type species. Anthracnose, caused by the *Colletotrichum* species, is a major disease of plants, mainly fruit, worldwide. It causes significant yield losses and reduces the marketability of the fruit [26,27,28,29]. There is growing evidence showing that *Colletotrichum* spp. is ubiquitous and widespread. As one of the top ten plant pathogenic fungi in the world [30], many *Colletotrichum* species were isolated from diseased plants, e.g., *C. miaoliense* P.C. Chung & H.Y. Wu and *C. australianum* W. Wang et al. were isolated from anthracnose symptoms on strawberry and citrus fruits [31,32]. In addition, *Colletotrichum* spp. were often reported as endophytes, including from healthy leaves of *Bletilla ochracea* [33]. Species of *Colletotrichum* are occasionally found as saprobes [34,35]. Some species were also isolated simultaneously as an endophyte, pathogen, and saprobe [36,37]. As for *Colletotrichum* spp. from air, an unidentified and a known species were reported. *Colletotrichum* sp. was isolated from the air of Dhaka, Bangladesh by Sultana [24]. Lal also trapped propagules of *C. falcatum* in air and confirmed that this species infected healthy plants [25]. 

When we investigated the fungi diversity of air samples in the town of Mengla, Xishuangbanna, a new species of *Colletotrichum* was identified based on morphological characteristics and DNA sequence data from four loci, and we named it *C. menglaense*. Its pathogenicity to several fruits was tested and confirmed by re-isolating the fungus. 

## 2. Results

### 2.1. Phylogenetic Analysis

**Figure 1:**
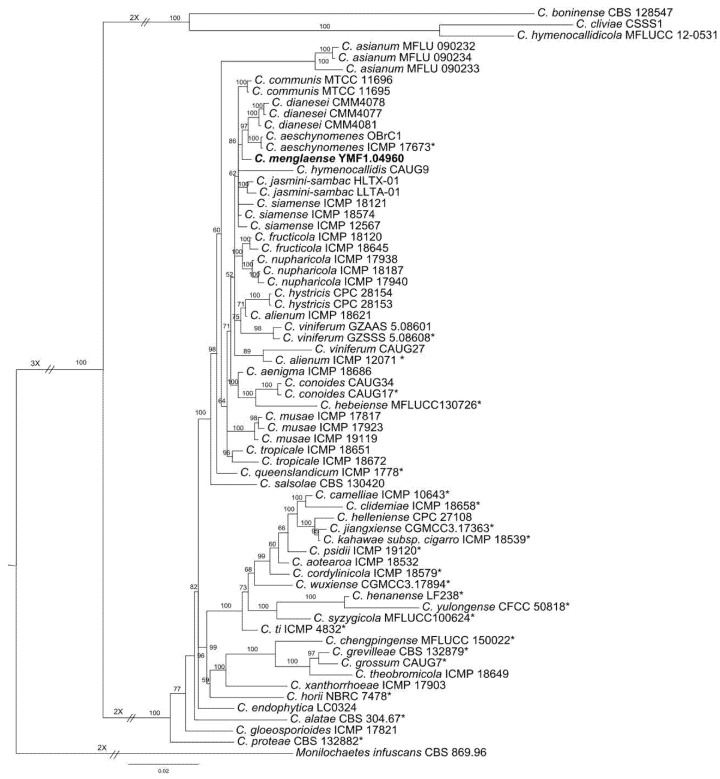
Phylogenetic tree based on Bayesian analysis of combined ITS, ACT, CHS, and GAPDH sequences. *Monilochaetes infuscans* was used as outgroup. Bayesian posterior probability values ≥ 0.85 were shown at the nodes. The scale bar shows the expected changes per site. * Ex-type strains. Newly described taxa were shown in boldface.

In the phylogenetic tree inferred from ITS, the strain is well clustered in the *Colletotrichum gloeosporioides* complex (not shown here). Therefore, we downloaded ITS, ACT, CHS, and GAPDH sequences in the *C. gloeosporioides* complex species. The dataset comprised 47 species, 67 isolates, and 1 outgroup taxa *Monilochaetes infuscans* (Table 1). A total of 1534 characters (ACT: 305, CHS: 300, GAPDH: 306, ITS: 623) were analysed by using Bayesian. The topologies of the tree were shown with the Bayesian posterior probability values for the analysed clades (Figure 1). In this tree, *C. menglense* is a sister clade to *C. aeschynomenes* B.S. Weir & P.R. Johns and *C*. *dianesei* N.B. Lima, M.P.S. Câmara & Michereff, and formed a single clade with high Bayesian inference posterior probability values (Figure 1). Therefore, we determined that our strain belonged to a novel species of *Colletotrichum*.

### 2.2. Pathogenicity Test

**Figure 2:**
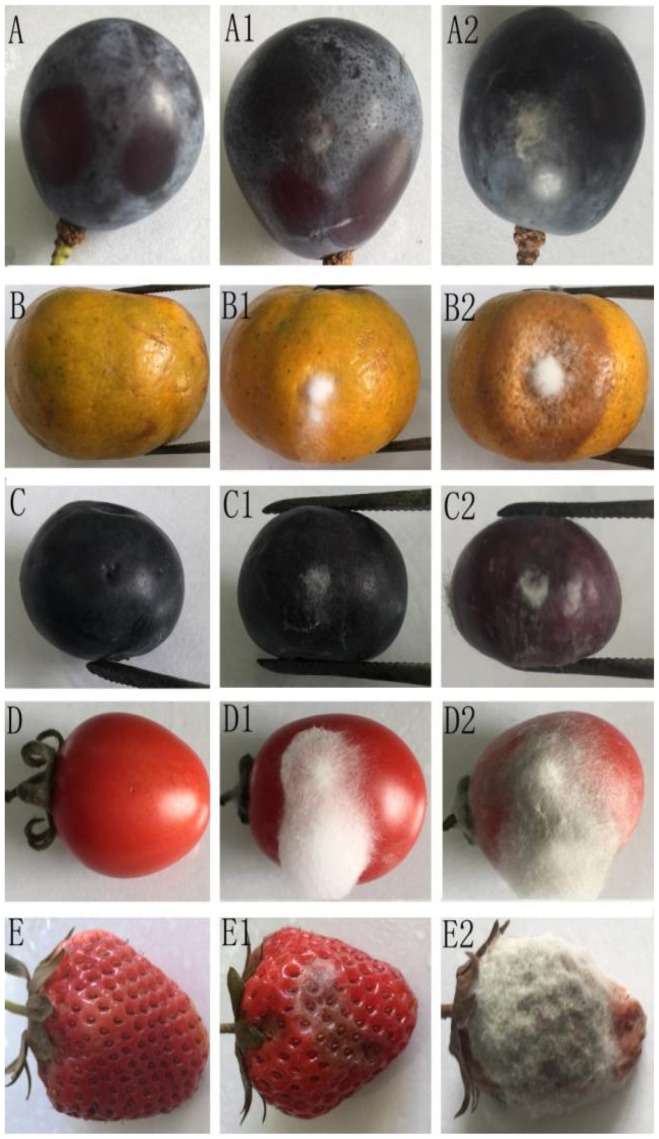
Pathogenicity test results of *C. menglaense*. (**A**–**E**). Control fruit. (**A1**–**E1**). Symptoms on fruits 7 d after inoculation. (**A2**–**E2**). Symptoms on fruits 14 d after inoculation.

After 7 days, five kinds of fruit inoculated with conidia suspension developed pale white hyphae around the wounds, and typical dark brown anthracnose lesions appeared on the strawberries, but no symptoms developed on the controls. Strawberry and tomato were the most susceptible, with disease scores from 7 to 9 (Table 2). Then, after 14 days, there were obvious anthracnose lesions around the wounds of the strawberry, orange, and tomato fruits. In fact, all of the fruits were susceptible to YMF 1.04960. The results showed that *C. menglaense* YMF 1.04960 is not host-specific (Figure 2). 

**Figure 3:**
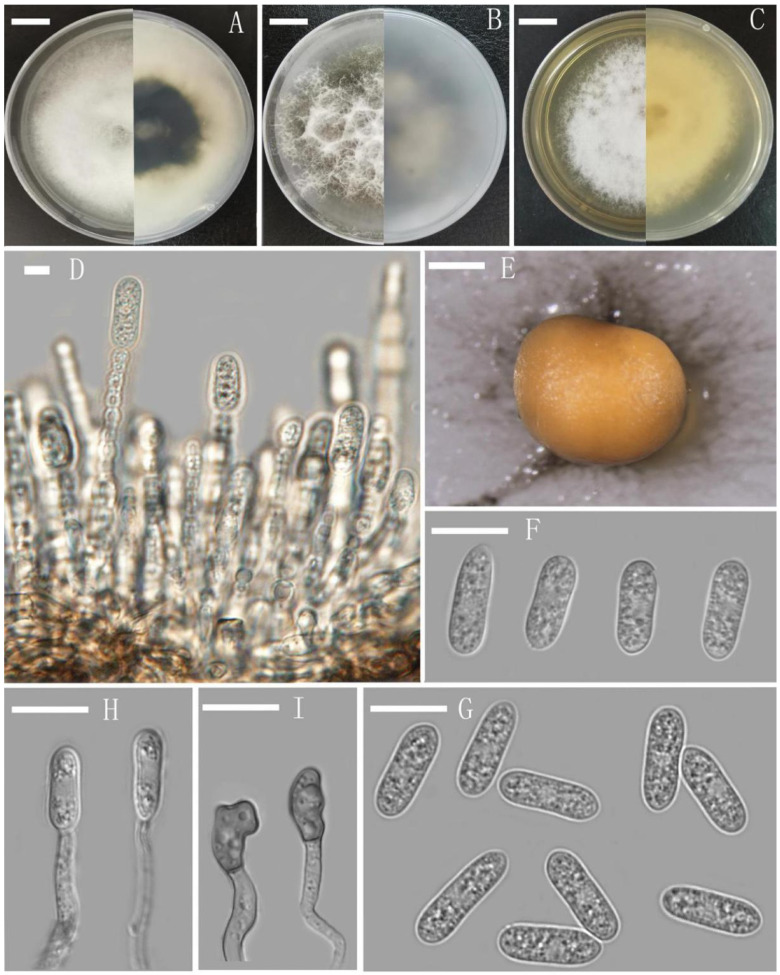
*Colletotrichum menglaense* (YMF1.04960). (**A**): Culture grown on PDA above and below (**B**): Culture grown on CMA above and below. (**C**): Culture grown on MEA above and below. (**D**,**H**): Conidiophores and conidia. (**E**): Conidioma on CMA. (**F**): Conidia from infected fruits. (**I**): Appressoria. (**G**): Conidia. Scale bars: (**A**–**C**) = 1.37 cm. (**D**) = 0.6 μm. (**E**) = 500 μm. (**F**,**G**) = 10 μm.

The conidia isolated from the infected fruits are the same as those of YMF 1.04960 (Figure 3F), and the ITS sequence is also the same as YMF 1.04960. So, the pathogenicity was confirmed.

### 2.3. Taxonomy

*Colletotrichum menglaense* M. Qiao & Z. F. Yu, sp. nov. -MycoBank: MB 839092; Figure 3.

Etymology: Latin, *menglaense,* referred to Mengla, the locality of the isolation in Yunnan Province.

Type: CHINA, Xishuangbanna Dai Autonomous Prefecture, Tropical Forest of Xishuangbanna Tropical Botanical Garden Chinese Academy of Sciences, 21°41’ N, 101°25’ E, ca 570 m, collected from the air, Jul 2016, Zefen Yu (holotype: YMF1.04960, ex-holotype: CGMCC 3.18958).

Description: Colonies growing on CMA with entire margin, 28–32 mm diameter in 4 d at 28 °C; aerial mycelia medium grey to pale buff in centre, light grey to greyish white in the margin, entirely covered with floccose to dense. Reverse dark white to grey with white margin. Conidiomata acervular, with orange conidial masses. No setae observed. Conidiophores cylindrical, unbranched or branched, straight or flexuous, 0–1-septate, hyaline, branched, 14.9–59.7 μm × 1.4–3.3 μm. Conidiogenous cells monophialidic, subulate, integrated, determinate, terminal, hyaline. Conidia acrogenous, oblong, sometimes slightly constricted at the middle, micro-guttulate, hyaline, unicellular, smooth-walled, 12.2–17.1 μm × 4.2–6.4 μm (av. = 14.4 μm × 5.1μm, *n* = 30). Appressoria simple, brown to dark brown, aseptate, mostly ellipsoidal to broadly obovoid, entire or irregular, somewhat crenate to lobed at margin, 6.7–20.0 μm × 4.8–11.0 μm, L/W ratio = 2.7.

Notes: *C. menglaense* can be distinguished from phylogenetically closely related *C. aeschynomenes* and *C. dianesei* by the dimensions of conidia. The conidia of *C. menglaense* are shorter (12.2–17.1 μm × 4.2 –6.4 μm, mean = 14.4 μm × 5.1 μm, *n* = 30) than those of *C. aeschynomenes* (14–)17–18.5 (–20) μm × 4 (–5) µm (mean = 17.6 μm × 4.1 µm, *n* = 30) [38] and longer and narrower than those of *C. dianesei* (10.5–14.5 μm × 4–5.5 µm, mean = 12.0 μm × 4.5 μm, *n* = 30) [39].

## 3. Materials and Methods

### 3.1. Sample Collection and Morphological Characterisation

Samples were collected from the Tropical Forest of Xishuangbanna Tropical Botanical Garden, Chinese Academy of Sciences (101°25′ E, 21°41′ N, altitude 570 m) in Mengla, Yunnan Province, China in July 2015. For each sample, we used a surface air system (SAS) Super ISO 180 (VWR European Cat.No.710-0870, San Giusto, Italy) that takes five minutes to capture 1,000 L of air. A 90 mm Petri dish containing RBA (5 g peptone, 10 g dextrose, 1 g potassium dihydrogen phosphate, 0.5 g magnesium sulfate, 15 g agar, 0.033 g rose bengal, 0.1 g chloramphenicol, 1000 mL distilled water) was put on the sampler for a few seconds to collect air. The Petri dishes were immediately sealed after air collection and brought back to the laboratory. Petri dishes were incubated in the continuous light at outdoor ambient temperature (mean 25 °C) and examined periodically. When a few mycelium appeared, it was picked up and transferred to PDA (200 g potato, 20 g glucose, 18 g agar, 40 mg streptomycin, 30 mg ampicillin, 1000 mL distilled water) dishes for incubation at 25 °C. The pure cultures were further incubated on CMA (20 g cornmeal, 18 g agar, 40 mg streptomycin, 30 mg ampicillin, 1000 mL distilled water) dishes to induce sporulation. Colony morphology and microscopic characteristics were examined, measured, and photographed after incubation for 7 days by using the aid of a BX51 microscope.

The pure culture was deposited in the Herbarium of the Laboratory for Conservation and Utilization of Bio-resources, Yunnan University, Kunming, Yunnan, China (YMF, formerly Key Laboratory of Industrial Microbiology) and the China General Microbiological Culture Collection Centre (CGMCC).

### 3.2. DNA Extraction, PCR Amplification, and Sequencing

Detailed protocols for Genomic DNA extraction are described previously [40,41]. The relative quantity of total genomic DNA was observed on a 1% TAE agarose gel stained with ethidium bromide. The following loci were amplified with the indicated primers: the internal transcribed spacer (ITS) region and actin gene (ACT) with primers ITS4/ITS5 [42,43]; ACT512F/ACT783R [40], respectively. The thermo-cycling parameters were used: initial denaturation at 95 °C for 3 min, followed by 34 cycles of 95 °C for 1 min, 52 °C for 30 s, 72 °C for 1 min, with a final extension step of 72 °C for 10 min. Chitin synthase 1 (CHS-1) were amplified with CHS-79F/CHS-354R [44]. The cycling parameters consisted of 94 °C for 5 min, followed by 35 cycles at 94 °C for 30 s, 56 °C for 30 s, 72 °C for 90 s, and a final extension step of 72 °C for 7 min. Partial sequences of the glyceraldehyde -3-phosphate dehydrogenase (GAPDH) were amplified with primers GDF1/GDR1 [45]. The cycling parameters consisted of a denaturation step at 94 °C for 4 min, followed by 34 cycles at 94 °C for 45 s, 60 °C for 45 s, 72 °C for 1 min, and a final cycle at 72 °C for 10 min. Amplification was performed in a total of 25 µL reaction volume, which contained 1.0 µL DNA template, 1.0 µL of each forward and reverses primer, 12.5 µL 2 × Master Mix (Tiangen Biotech, Beijing, China), and 9.5 µL dd H_2_O. The sequencing reactions were carried out by TsingKe Biological Technology, Kunming, China using the same primers as for amplification. The new sequences were submitted to the GenBank database at the National Center for Biotechnology Information (NCBI), and the accession numbers are listed in Table 1.

### 3.3. Phylogenetic Analysis

The obtained ITS sequences were compared with those in GenBank using BLAST searches to determine the primary phylogenetic placement of the fungus. The results indicated that our strain belongs to *Colletotrichum*. Neighbour-joining analysis of ITS sequence was used to determine further phylogenetic placement. Then, we retrieved ITS, ACT, GAPDH, and CHS sequences of representative species and additional species belonging to this complex species. All sequences used in this study are listed in Table 1 and were aligned through ClustalX 1.83(Institut de Genetique et de Biologie Moleculaire et Cellulaire (CNRS/INSERM/ULP), Illkirch-Graffenstaden, France) [46]. The resulting alignments were subsequently manually adjusted and linked by BioEdit version v. 7.0(Borland, Austin, TX, United States) [47]. To ensure that all sequences are of the same length, the missing base was replaced with “?”. Then, the combined alignment was converted to a NEXUS file using the program mega7(Mega Limited, Auckland, New Zealand) [48]. Phylogenetic analyses were performed for Bayesian inference (BI) analysis using MrBayes v.3.2.2 (Department of Biodiversity Informatics, Swedish Museum of Natural History, Stockholm, Sweden) [49].

For BI analysis, the best nucleotide substitution model for each locus was determined using Mrmodeltest v. 2.3 (Department of Systematic Zoology, Evolutionary Biology Centre, Uppsala University, Uppsala, Sweden) [50]. The analyses of four MCMC chains were run from random trees for 1,000,000 generations, and trees were sampled every 100 generations, resulting in 20,000 total trees. The first 25 % of the trees were discarded as the burn-in phase of each analysis, and the remaining trees were used to calculate posterior probabilities. Sequences derived in this study were deposited in GenBank (Table 1), and the concatenated alignments were deposited in TreeBASE with http://purl.org/phylo/treebase/phylows/study/TB2:S27941, and the descriptions and nomenclature in MycoBank (www.mycobank.org).

### 3.4. Pathogenicity Assay and Confirmation

In order to test the pathogenicity of the new species, detached fruits inoculations were conducted. Healthy *Fragaria ananassa* Duch. (strawberry), *Citrus tachibana* (Makino) Tanaka (orange), *Vitis vinifera* L. (grape), *Lycopersicon esculentum* Mill. (tomato), and *Vaccinium uliginosum* Linn. (blueberry) were used for the pathogenicity test. All fruits were immersed in 70% ethanol for 3 min and 1% sodium hypochlorite for 3 min, then rinsed three times in sterile distilled water and air dried in the laminar flow cabinet.

Prior to the inoculation, holotype strain YMF 1.04960 of new species was cultivated on CMA for 7 days at 28 °C, adding 0.4 g yeast extract per 100 mL to induce sporulation. After incubation, conidia were harvested by adding 10 mL sterile water to each culture followed by scraping the surface with a sterile brush. The resulting conidia suspensions were filtered through sterile six layers of filter paper. Then, conidia were diluted to 10^6^/mL using sterile water (concentration was adjusted by using a haemocytometer). Fruit were wounded with a sterilised insect needle and inoculated with 10 μL conidium suspension. Control fruits were inoculated with sterilised water. Five replications were set. The inoculated fruits with the controls were put into plastic containers, covered with plastic wrap to maintain humidity, sealed and stored in a constant temperature incubator, and examined periodically. 

Seven days and 14 days after inoculation, the virulence was evaluated as described by Montri et al. [51]. In particular, 0 (highly resistant), no infection; 1 (resistant), 1–2% of the fruit with a necrotic lesion or a larger water soaked lesion surrounding the infection site; 3 (moderately resistant), >2 to 5% of the fruit with a necrotic lesion, possibly acervuli, may be present, or a watery lesion covering up to 5% of the fruit surface; 5 (susceptible), >5 to 10% of the fruit showing a necrotic lesion, possibly acervuli, or a water-soaked lesion covering up to 25% of the fruit surface; 7 (very susceptible), >10 to 25% of the fruit covered with a necrotic lesion with acervuli; and 9 (highly susceptible), >25% of the fruit showing necrosis, lesion often encircling the fruit, abundant acervuli. Symptomatic fruits were surface-sterilised as described above. The symptomatic tissue segments were cut with a sterilised scalpel about 5 mm × 5 mm × 5 mm and then placed on the PDA to re-isolate the fungus. The identity of obtained isolates was confirmed on the basis of morphological characteristics and ITS sequence. 

## 4. Discussion

ITS has been proposed as the official fungal barcoding marker [52], but phylogenetic analysis using only ITS sequences could not confidently resolve the phylogenetic placement of some species within *Colletotrichum*. In this study, phylogenetic analysis based on ITS showed that *C. menglaense* could not be distinguished from *C. queenslandicum*, *C. salasolae,* and *C. siamense,* but the results showed that *C. menglaense* is well clustered in the *C. gloeosporioides* species complex, so we further used multi-locus phylogeny to distinguish closely related species. Combining sequences of ITS, ACT, CHS, and GAPDH, the phylogenetic position of *C. menglaense* was determined. In the phylogenetic tree, the species relationships were well defined, with all of the major clades supported by high Bayesian inference posterior probabilities (Figure 1). *C. menglaense* grouped together with *C. aeschynomenes* and *C. dianesei*, and the ITS similarity between *C. menglaense* and *C. aeschynomenes* (KU239115) is 99.08%, while, between *C. menglaense* and *C. dianesei* (KC329775), it is 99.47%. Morphologically, *C. menglaense* obviously differs from *C. aeschynomenes* and *C. dianesei* in the shape and size of the conidia.

The pathogenicity test showed that *C. menglaense* may be a potential pathogen to fruit. Among all of the test fruits, *C. menglaense* was very aggressive on strawberries, while it was less aggressive on blueberries and grapes. This is in agreement with Xavier et al., who reported that the *C. gloeosporioides* species complex was more aggressive to strawberry than other *Colletotrichum* species complex organisms [53]. The degree of fruit infection may be related to fruit condition, humidity, temperature, inoculum concentration, and inoculation method [54], so, among fruits tested here, strawberry with softer tissue showed the highest disease scores of 5 to 9. Several fruits inoculated with *C. menglaense* presented different degrees of anthracnose, indicating that *C. menglaense* was non-host specific. In fact, many *Colletotrichum* species present on a wide range of host plants [27,55]. For example, *C. karstiiwas* was reported from diseased black plum (*Diospyros australis*), strawberry (*Fragaria xananassa*), and banana (*Musa nana Lour*). *C. gloeosporioides* species complex organisms were also frequently isolated from a variety of hosts, including kumquat, finger lime, grapefruit, lemon, lime, mandarin, orange, and Persian lime [37,56].

Previously, it was reported that *Cladosporium* was the most frequent fungus in the air; the next were *Fusarium*, *Alternaria,* and *Epicoccum* [57]. Some leaf surface fungi are major contributors to air spores through the action of wind or rain spatter, and the canopy is closer to the leaves of the plant, so there are more fungal spores in the air below the canopy [58]. Similar to previous reports, the air samples that we obtained were collected in the lower part of the canopy. Besides, some airborne spores have been reported to be pathogenic fungi. *Alternaria alternata* airborne spores might be sufficient to cause human spore-related asthma symptoms to people even with only a limited concentration [59]. Here, *C. menglaense* is an airborne fungus that has certain pathogenicity to plants. Previous studies also showed that *Colletotrichum* spp. from air were pathogenic fungi to plants [25]. Due to limited study, we do not know how many pathogenic fungi are present in the air and how they contribute to the spread of plant diseases. In this respect, the present article provides new information.

## Figures and Tables

**Table 1:** Fungal strains and the GenBank accession numbers of their sequences used in molecular phylogenetic analyses in this study.

Name of the Taxon	Culture Collection	GenBank Accessions Numbers			
		ACT	ITS	CHS	GAPDH
*C. aenigma*	ICMP 18686	JX009519	JX010243	JX009789	JX009913
*C. aeschynomenes*	OBrC1	KU239794	KU239115	KU239352	KU239576
*C. aeschynomenes*	ICMP 17673 *	JX009483	JX010176	JX009799	JX009930
*C. alatae*	CBS 304.67 *	JX009471	JX010190	JX009837	JX009990
*C. alienum*	ICMP 18621	JX009552	JX010246	JX009755	JX009959
*C. alienum*	ICMP 12071 *	JX009572	JX010251	JX009882	JX010028
*C. aotearoa*	ICMP 18532	JX009544	JX010220	JX009764	JX009906
*C. asianum*	MFLU 090232	FJ903188	FJ972605		FJ972571
*C. asianum*	MFLU 090234	FJ907421	FJ972615		FJ972573
*C. asianum*	MFLU 090233	FJ907424	FJ972612		FJ972576
*C. boninense*	CBS 128547	JQ005507	JQ005159	JQ005333	JQ005246
*C. camelliae*	ICMP 10643 *	JX009540	JX010224	JX009891	JX009908
*C. chengpingense*	MFLUCC 150022 *	KP683093	KP683152	KP852449	KP852469
*C. clidemiae*	ICMP 18658 *	JX009537	JX010265	JX009877	JX009989
*C. cliviae*	CSSS1	GU085861	GU109479	GU085865	GU085867
*C. communis*	MTCC 11696	KF451940	KC790977	KF451988	KF452016
*C. communis*	MTCC 11695	KF451941	KC790980	KF451989	KF452017
*C. conoides*	CAUG34	KP890146	KP890170	KP890158	KP890164
*C. conoides*	CAUG17 *	KP890144	KP890168	KP890156	KP890162
*C. cordylinicola*	ICMP 18579 *	HM470235	JX010226	JX009864	JX009975
*C. dianesei*	CMM4078	KC533745	KC329775		KC517158
*C. dianesei*	CMM4081	KC517304	KC329790		KC517166
*C. dianesei*	CMM4077	KC517295	KC329773		KC517156
*C. endophyticum*	LC0324	KF306258	KC633854		KC832854
*C. fructicola*	ICMP 18120	JX009436	JX010182	JX009844	JX010041
*C. fructicola*	ICMP 18645	JX009543	JX010172	JX009873	JX009992
*C. gloeosporioides*	ICMP 17821	JX009531	JX010152	JX009818	JX010056
*C. grevilleae*	CBS 132879 *	KC296941	KC297078	KC296987	KC297010
*C. grossum*	CAUG7 *	KP890141	KP890165	KP890153	KP890159
*C. hebeiense*	MFLUCC130726 *	KF377532	KF156863	KF289008	KF377495
*C. helleniense*	CPC 27108	KY856022	KY856449	KY856189	KY856273
*C. henanense*	LF238 *	KM023257	KJ955109		KJ954810
*C. horii*	NBRC 7478 *	JX009438	GQ329690	JX009752	GQ329681
*C. hymenocallidicola*	MFLUCC 12-0531	KT290260	KT290264	KT290262	
*C. hymenocallidis*	CAUG9	KP145311	KP145423	KP145367	KP145395
*C. hystricis*	CPC 28154	KY856024	KY856451	KY856191	KY856275
*C. hystricis*	CPC 28153	KY856023	KY856450	KY856190	KY856274
*C. jasmini-sambac*	HLTX-01		HM131512		HM131498
*C. jasmini-sambac*	LLTA-01	HM131507	HM131511		HM131497
*C. jiangxiense*	CGMCC3.17363 *	KJ954471	KJ955201		KJ954902
*C. cigarro*	ICMP 18539 *	JX009523	JX010230	JX009800	JX009966
***C. menglaense***	**YMF1.04960**	**MH023506**	**MH023505**	**MH023508**	**MH023507**
*C. musae*	ICMP 17817	JX009432	JX010142	JX009815	JX010015
*C. musae*	ICMP 19119	JX009433	JX010146	JX009896	JX010050
*C. musae*	ICMP 17923	JX009587	JX010143	JX009841	JX009929
*C. nupharicola*	ICMP 17938	JX009486	JX010189	JX009834	JX009936
*C. nupharicola*	ICMP 18187	JX009437	JX010187	JX009835	JX009972
*C. nupharicola*	ICMP 17940	JX009582	JX010188	JX009836	JX010031
*C. proteae*	CBS 132882 *	KC296940	KC297079	KC296986	KC297009
*C. psidii*	ICMP 19120 *	JX009515	JX010219	JX009901	JX009967
*C. queenslandicum*	ICMP 1778 *	JX009447	JX010276	JX009899	JX009934
*C. salsolae*	CBS 130420	JX009562	JX010242	JX009863	JX009916
*C. siamense*	ICMP 18121	JX009460	JX010245	JX009845	JX009942
*C. siamense*	ICMP 18574	JX009535	JX010270	JX009798	JX010002
*C. siamense*	ICMP 12567	JX009541	JX010250	JX009761	JX009940
*C. syzygicola*	MFLUCC100624 *	KF157801	KF242094		KF242156
*C. theobromicola*	ICMP 18649	JX009444	GU994360	JX009869	JX010006
*C. ti*	ICMP 4832 *	JX009520	JX010269	JX009898	JX009952
*C. tropicale*	ICMP 18651	JX009570	JX010277	JX009868	JX010014
*C. tropicale*	ICMP 18672	JX009480	JX010275	JX009826	JX010020
*C. viniferum*	GZAAS 5.08601	JN412795	JN412804		JN412798
*C. viniferum*	GZSSS 5.08608 *	JN412793	JN412802		JN412800
*C. viniferum*	CAUG27	KP145328	KP145440	KP145384	KP145412
*C. wuxiense*	CGMCC3.17894 *	KU251672	KU251591	KU251939	KU252045
*C. xanthorrhoeae*	ICMP 17903	JX009478	JX010261	JX009823	JX009927
*C. yulongense*	CFCC 50818 *	MH777394	MH751507	MH793605	MK108986
*Monilochaetes infuscans*	CBS 869.96	JQ005843	JQ005780	JQ005801	JX546612

Abbreviations of isolates and culture collections: MFLU: Mae Fah Luang University, Thailand; CMM: Culture Collection of Phythopathogenic Fung “Prof. Maria Menezes”, Recife, Brazil; ICMP: International Collection of Micro-organisms from Plants, Landcare Research, New Zealand; MAFF: Ministry of Agriculture, Forestry and Fisheries, Tsukuba, Japan; MTCC: Microbial Type Culture Collection and Gene Bank, Chandigarh, India; MFLUCC: The Mae Fah Luang University Culture Collection; CPC: Culture collection of P.W. Crous, housed at the Westerdijk Institute; CBS: Westerdijk Fungal Biodiversity Institute, Utrecht, The Netherlands; YMF: formerly Key Laboratory of Industrial Microbiology and Fermentation Technology of Yunnan. * Ex-type strains. Sequences obtained in this study were shown in bold.

**Table 2:** Disease Score (DS) on a 0–9 scale of different fruits for *C. menglaense* inoculated by wounding or non-wounding methods.

Number of Days	Fruit				
	Strawberry	Orange	Grape	Tomato	Blueberry
7	7	3	1	9	3
14	9	9	3	9	9

## Data Availability

All sequences have been deposited in GenBank at the accession numbers given in the text. The GenBank accession numbers of *Colletotrichum menglaense*: ITS MH023505; ACT MH023506; GAPDH MH023507; CHS MH023508.

## Abbreviations

RBA: Rose Bengal Agar; PDA, potato dextrose agar; CMA, corn meal agar; ITS, internal transcribed spacer; ACT, actin gene; CHS-1, chitin synthase 1; GAPDH, the glyceraldehyde-3-phosphate dehydrogenase.
